# Supplementary material for: Using pose estimation to identify regions and points on natural history specimens
Source: PLoS Comput Biol. 2023 Feb 22;19(2):e1010933. doi: 10.1371/journal.pcbi.1010933 (PMC9987800; doi:10.1371/journal.pcbi.1010933)
Supplement: S1 Appendix — (DOCX) [file pcbi.1010933.s001.docx]

Using pose estimation to identify regions and points on natural history specimens

**Authors:** Yichen He^1^*, Christopher R. Cooney^1^, Steve Maddock^2^, Gavin H. Thomas^1,3^

**Affiliations:**

^1^ Ecology and Evolutionary Biology, School of Biosciences; Alfred Denny Building, University of Sheffield, Western Bank, Sheffield S10 2TN, UK.

^2^ Department of Computer Science, University of Sheffield; Regent Court, University of Sheffield, 211 Portobello, Sheffield S1 4DP, UK.

^3^ Bird Group, Department of Life Sciences, The Natural History Museum at Tring; Akeman Street, Tring, HP23 6AP, UK.

*Corresponding authors. Email: csyichenhe@gmail.com

**Network architectures & resolutions**

We compared two network architectures: Stacked Hourglass and Convolutional pose machine (CPM)(1) with different image resolutions. Specifically, we used resolutions that were 10, 15, and 20 times lower than the input images (i.e. 494 x 328, 329 x 218 and 247 x 164 pixels). This image resolution manipulation gives five comparisons against the benchmark configuration (Stacked Hourglass, 494 x 328 pixels) described in the main text.

We found that there were significant effects on pixel distances depending on architecture and resolution both overall and for individual points (S3 Table). The Stacked Hourglass method with the highest pixel resolution had the best performance (S3a Fig). Pixel distances of the Stacked Hourglass model were significantly better (i.e. smaller) than CPM under the same input resolution. The mean difference of pixel distances (of all and individual points) between two networks was 29.5 (95% CI: 28.8 and 30.2; t(242973) = 86.9, p<0.0001) under the resolution of 4948 x 3280 pixels (about 0.9% of the image height). The input resolution was positively related to performance. The result using 494 x 328 pixels scored the lowest pixel distance, and was significantly better than the result using 329 x 218 pixels (mean difference: 4.1; 95% CI: 3.5 and 4.8; t(168503) = 12.6, p<0.0001) and 247 x 164 pixels (mean difference: 36.0; 95% CI: 35.1 and 36.8; t(138705) = 79.4, p<0.0001).

Overall, among the six trained configurations, the CPM network with 247 x 164 pixels input images had the largest pixel distance between the ground truth and model predictions and the Stacked Hourglass with 494 x 328 has the lowest pixel difference. The best configuration inferred by pixel distance (the Stacked Hourglass with 494 x 328) can predict all standards inside reflectance standards as PCK-100 of the reflectance standards 1-5 are 100% (S3b Fig) and ground truth points were always placed in standard centres and the minimum radius was larger than 100 pixels. The predictions from the Stacked Hourglass with 494 x 328 pixels images had the highest overall and per-region colour correlation coefficients (S3c Fig).

**Pose estimation performance with low-quality datasets**

All avian specimen images were taken in a highly consistent manner by controlling the placement of the specimen, light environment and background (2). Not all datasets are likely to be so consistent. We therefore tested whether greater variability in data quality could limit performance by generating lower quality datasets. To do this, we applied a series of affine transformations to the images and their labels as well. Four datasets were created with different transformations applied: (i) rotation (angles between -45° to 45°), (ii) translation on both x and y axes (-500 to 500 pixels), (iii) horizontal flip 50% images randomly, (iv) the combination of all three transformations. 45 degrees rotation and 500 pixels translation give images large transformations while keeping all points inside the image. The transformed datasets are trained and evaluated with the model of Stacked Hourglass, the input resolution of 494 x 328 pixels and the training duration of 15 epochs.

**Human error checking**

308 regions were predicted incorrectly among the 234 images (165 images had one error; 64 images had two errors; 5 images had three errors). Flight feathers, coverts, tail, rump and crown were the five most problematic regions (S2 Fig), which is consistent with the higher pixel distances and lower PCK100 scores and colour metric correlations for these body regions. One expert (YH) went through the 5094 images, and flagged images that he think would be difficult to label manually without knowing the predictions. 135images were flagged as difficult-to-label. The obstacles were categorised into four causes, (i) small body region (47 images), (ii) similar colour to the adjacent area (36 images), (iii) rare posture of the specimen (38 images) and (iv) partially occluded body region (14 images). 32 images were both incorrectly labelled (234 images) and difficult-to-label (135 images). The 32 images consisted of 12 with small body regions, 7 with similar colour, 11 with rare postures and 2 with partially occluded regions. Notably, incorrectly predicted flight feathers and coverts tend to associate with features of small area and similar colour, and the majority of incorrectly predicted tails are partially occluded.

**References**

1. Wei S-E, Ramakrishna V, Kanade T, Sheikh Y. Convolutional pose machines. In: Proceedings of the IEEE Conference on Computer Vision and Pattern Recognition. 2016.

2. Blagoderov V, Kitching IJ, Livermore L, Simonsen TJ, Smith VS. No specimen left behind: Industrial scale digitization of natural history collections. Zookeys. 2012;209:133–46.
