# Supplementary figures and images for: Using pose estimation to identify regions and points on natural history specimens
Source: PLoS Comput Biol. 2023 Feb 22;19(2):e1010933. doi: 10.1371/journal.pcbi.1010933 (PMC9987800; doi:10.1371/journal.pcbi.1010933)

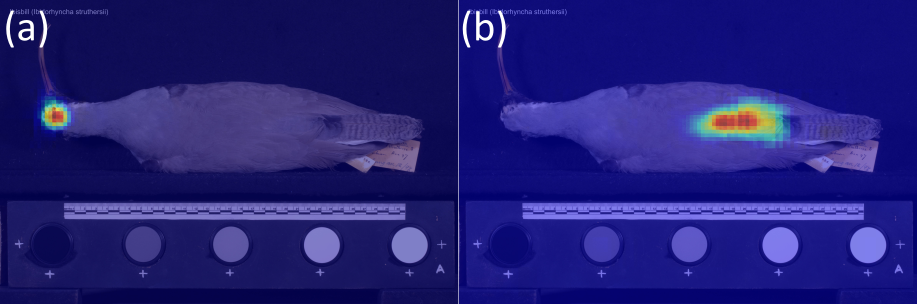

Supplement: S1 Fig — Heatmaps are from two body regions on a representative bird specimen image. (a) The predicted heatmap of the crown has a smaller area than the one of the (b) rump which has an ellipse-like shape and can capture more area of the rump region than using a fixed-size area. (TIF) [file pcbi.1010933.s002.tif]

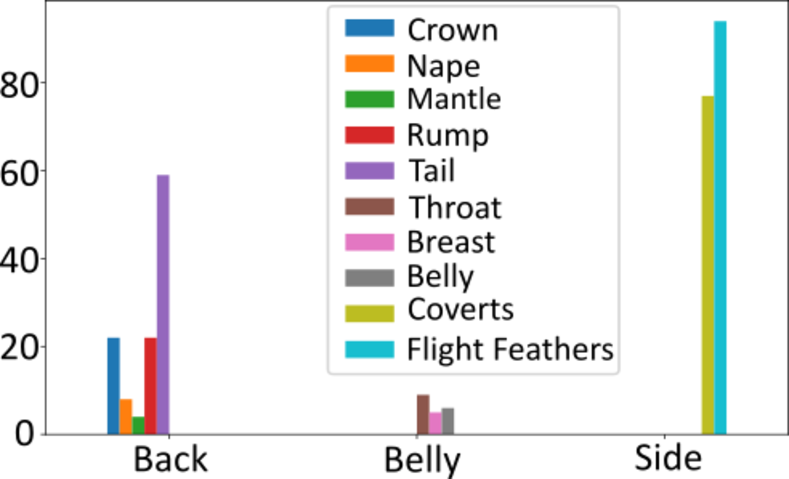

Supplement: S2 Fig — Error prediction counts (N = 308) for each body region. (TIF) [file pcbi.1010933.s003.tif]

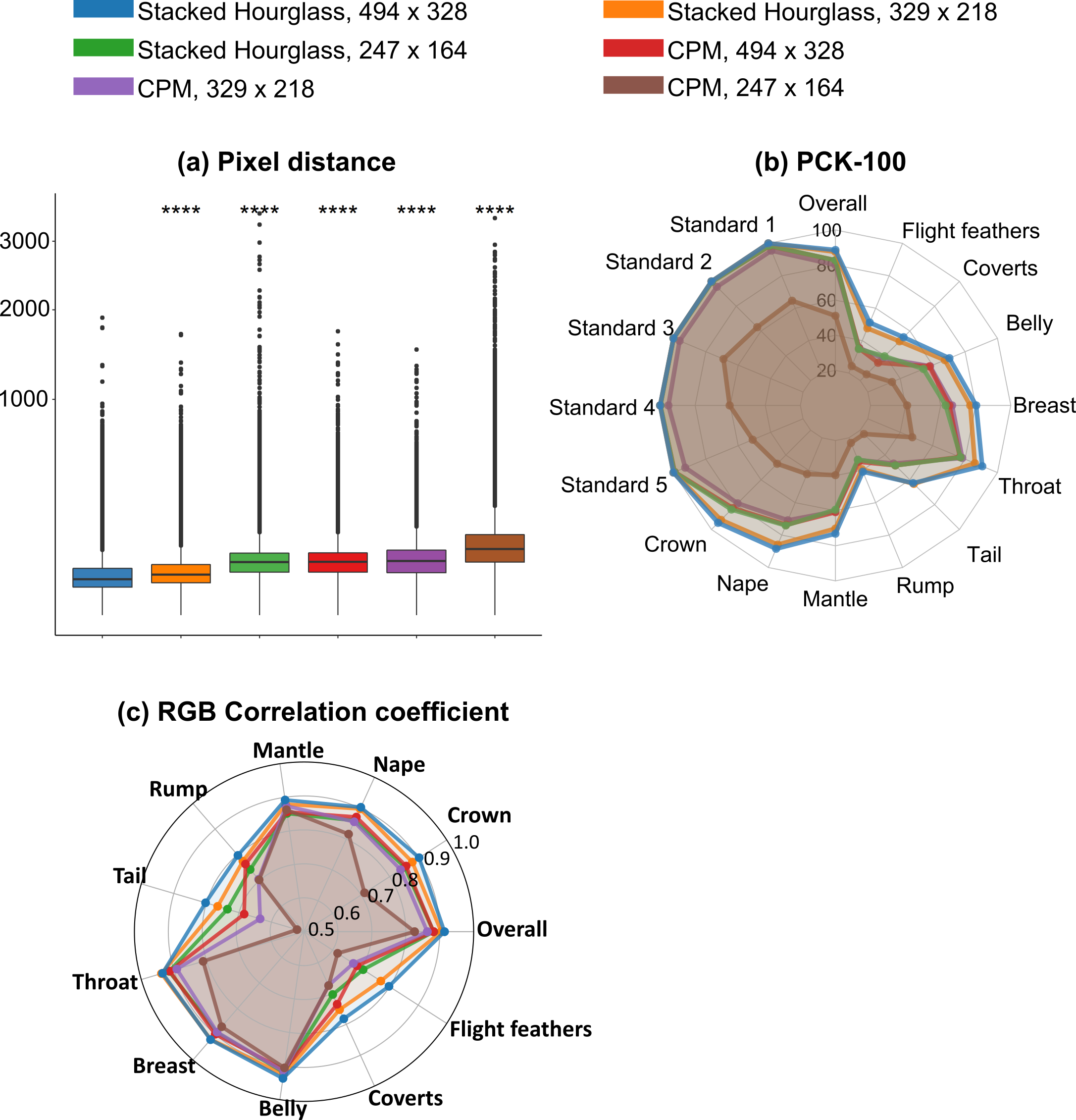

Supplement: S3 Fig — The plots show comparisons of model performance by comparing metrics from the ground truth data with the model prediction: (a) Pixel distances. The Y axis (pixel distance) is square root scaled; (b), PCK-100 of all and individual points; (c) RGB colour correlation coefficients of all and individual point-defined body regions (colour extraction method: Bbox-20). (TIF) [file pcbi.1010933.s004.tif]

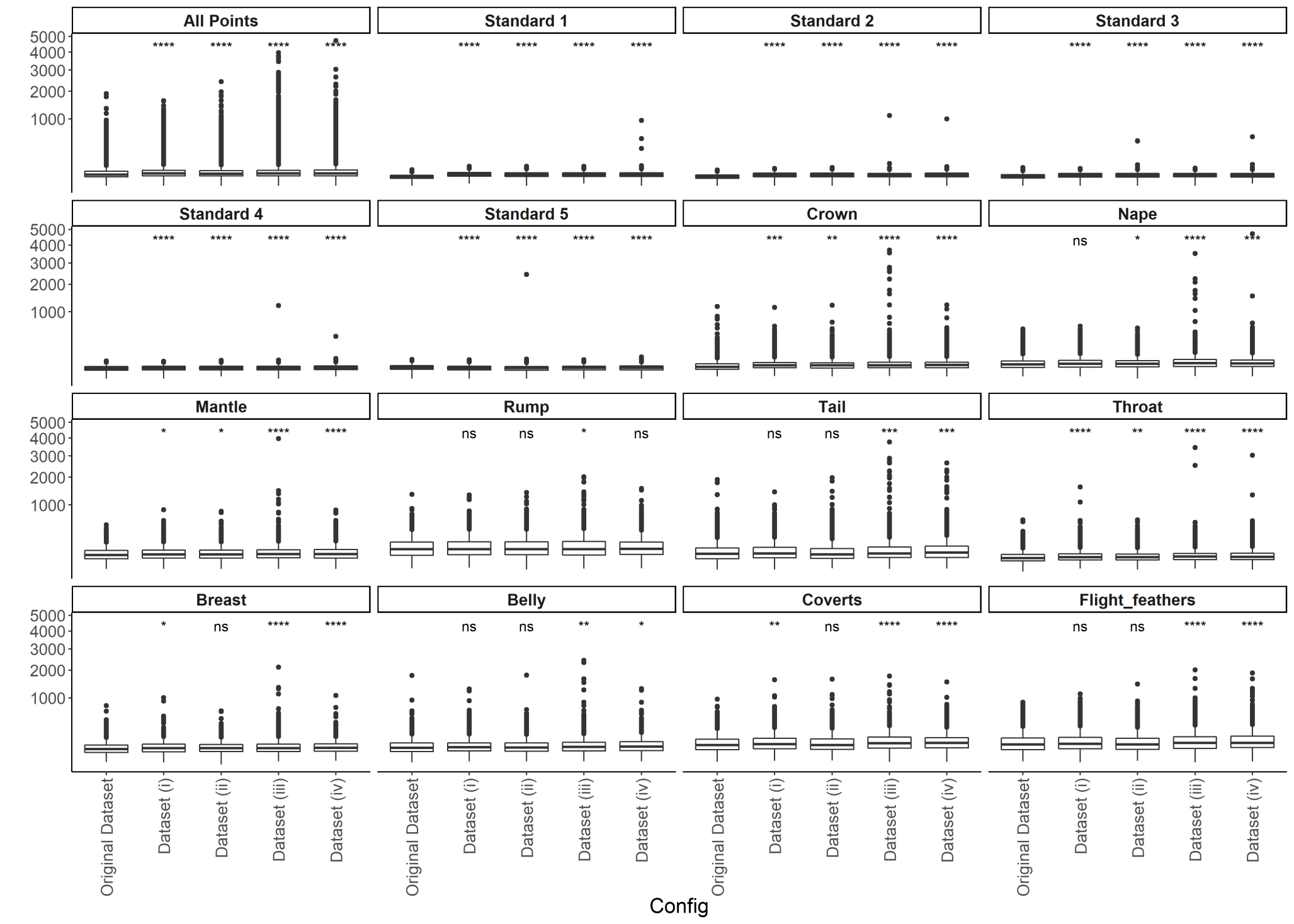

Supplement: S4 Fig — The average pixel distance (the top left plot) of the original dataset is significantly smaller than the pixels distances of the low-quality datasets (ns: p > 0.05; *: p < = 0.05; **: p < = 0.01; ***: p < = 0.001; ****: p < = 0.0001). The four low-quality datasets are: (i) rotation (angles between -45° to 45°), (ii) translation on both x and y axes (-500 to 500 pixels), (iii) horizontal flip 50% images randomly, (iv) the combination of all three transformations. The Y axes (pixel distance) are square root scaled. (TIF) [file pcbi.1010933.s005.tif]

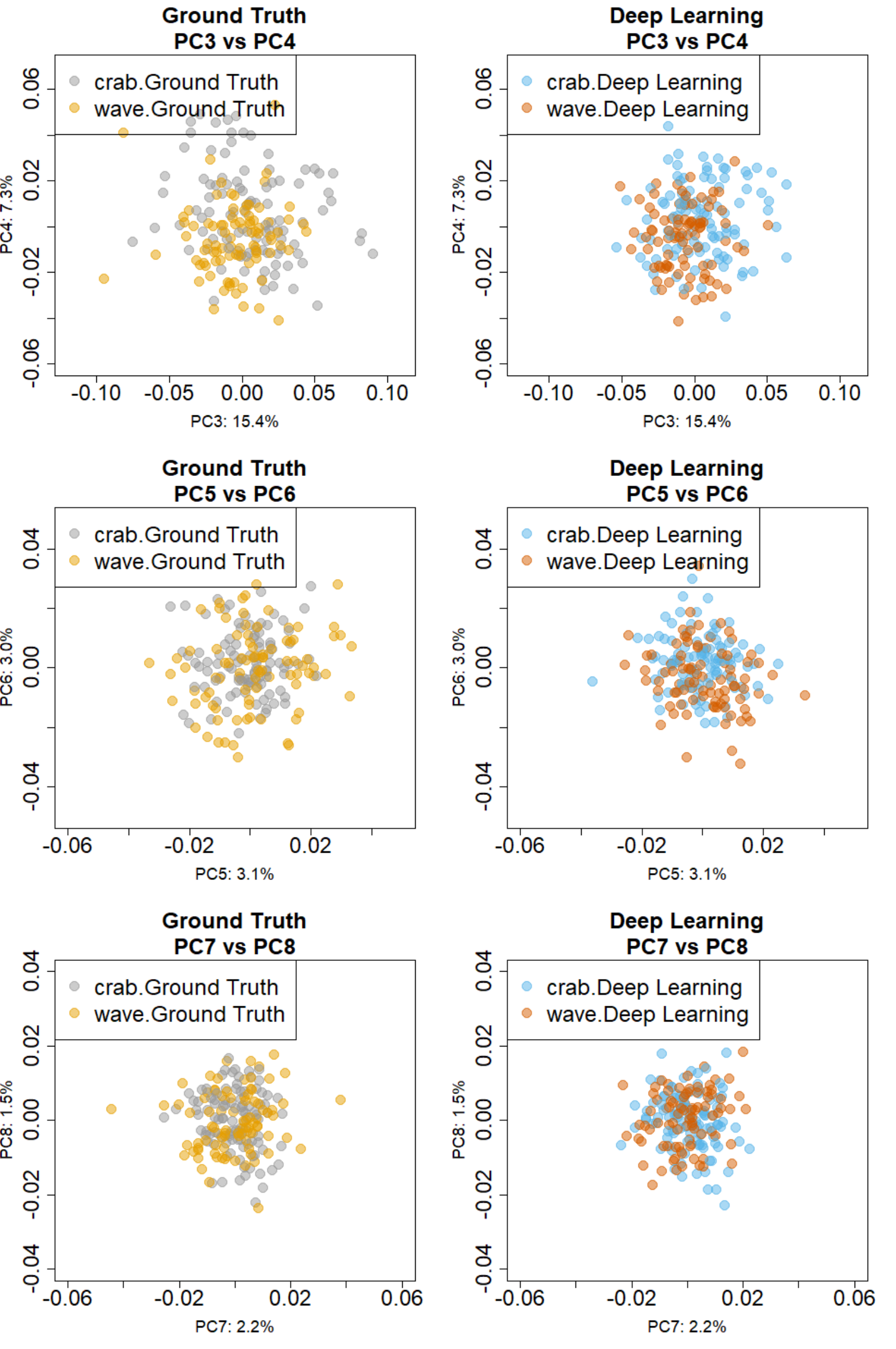

Supplement: S5 Fig — PC3-8 explain 32.4% of the total variation. Left: the ground truth landmarks (Crab: Grey; Wave: Yellow). Right: Deep Learning predicted landmarks (Crab: Blue; Wave: Red). (TIF) [file pcbi.1010933.s006.tif]

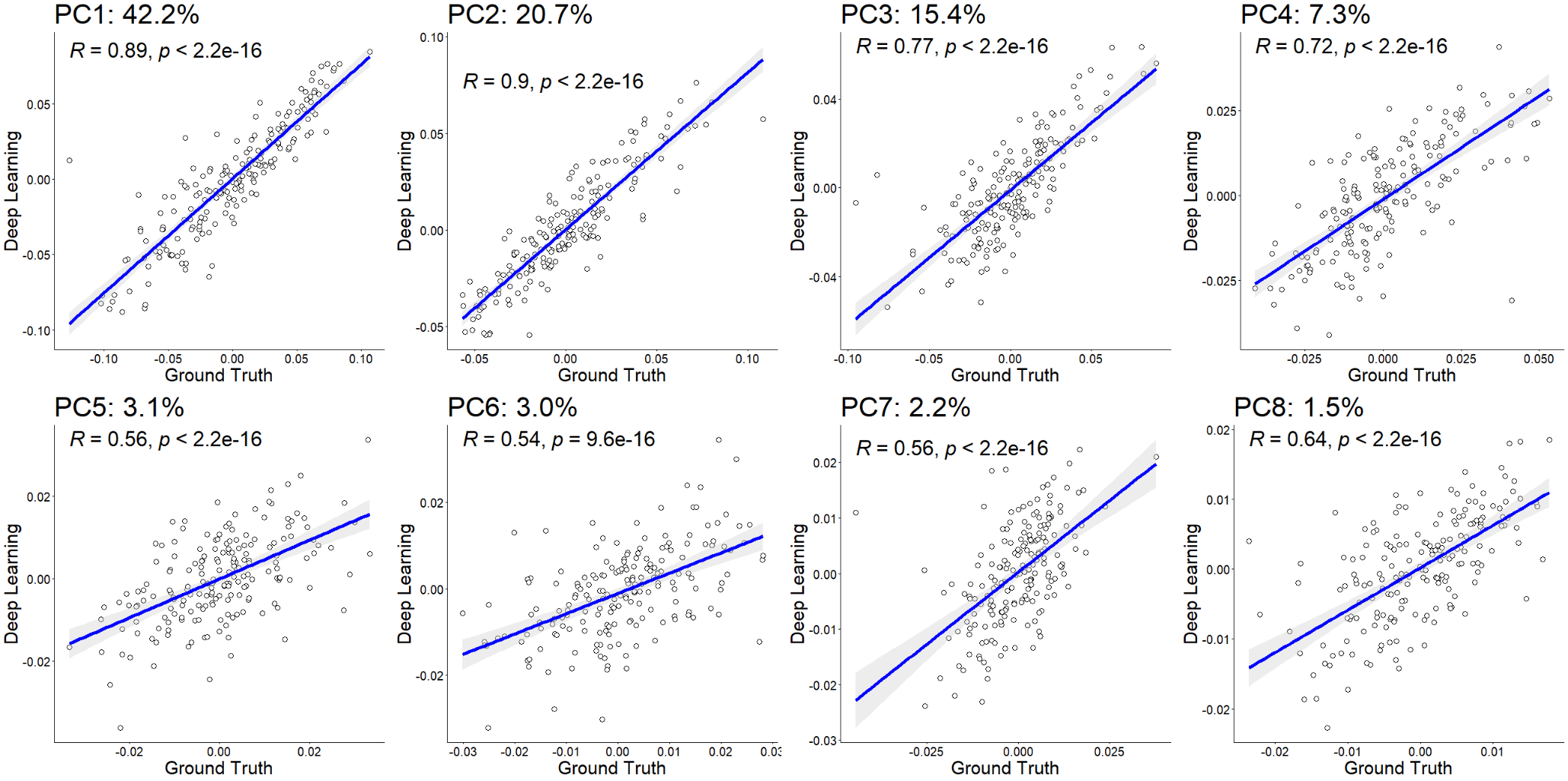

Supplement: S6 Fig — (TIF) [file pcbi.1010933.s007.tif]
