# Supplementary material for: Using pose estimation to identify regions and points on natural history specimens
Source: PLoS Comput Biol. 2023 Feb 22;19(2):e1010933. doi: 10.1371/journal.pcbi.1010933 (PMC9987800; doi:10.1371/journal.pcbi.1010933)
Supplement: S1 Table — The datasets are (a) the full dataset; (b) the genus-level dataset; (c) the imbalanced training set and (d) the imbalanced test set. (PDF) [file pcbi.1010933.s008.pdf]

S1 Table. **Tables of images counts and proportions for each order in four datasets.** The datasets are (a) the full dataset; (b) the genus-level dataset; (c) the imbalanced training set and (d) the imbalanced test set.

	Number of images	Proportion (%)
**(a) The full dataset**	234,954	
Accipitriformes	5778	2.46
Apodiformes	10872	4.63
Bucerotiformes	1650	0.7
Caprimulgiformes	2478	1.05
Charadriiformes	11058	4.71
Ciconiiformes	498	0.21
Coliiformes	216	0.09
Columbiformes	7788	3.31
Coraciiformes	4638	1.97
Cuculiformes	4092	1.74
Eurypygiformes	54	0.02
Falconiformes	1944	0.83
Galliformes	246	0.1
Gruiformes	3462	1.47
Leptosomiformes	36	0.02
Mesitornithiformes	54	0.02
Musophagiformes	768	0.33
Opisthocomiformes	30	0.01
Otidiformes	660	0.28
Passeriformes	154896	65.93
Pelecaniformes	2808	1.2
Piciformes	12444	5.3
Procellariiformes	2574	1.1
Pteroclidiformes	576	0.25
Sphenisciformes	396	0.17
Strigiformes	3750	1.6
Trogoniformes	1188	0.51
**(b) The genus-level dataset**	5094	
Accipitriformes	195	3.83
Apodiformes	363	7.13
Bucerotiformes	42	0.82
Caprimulgiformes	48	0.94
Charadriiformes	246	4.83
Ciconiiformes	9	0.18
Coliiformes	6	0.12
Columbiformes	120	2.36
Coraciiformes	96	1.88
Cuculiformes	66	1.3
Eurypygiformes	3	0.06
Falconiformes	33	0.65

Galliformes	15	0.29
Gruiformes	111	2.18
Leptosomiformes	3	0.06
Mesitornithiformes	6	0.12
Musophagiformes	15	0.29
Opisthocomiformes	3	0.06
Otidiformes	6	0.12
Passeriformes	3408	66.9
Pelecaniformes	6	0.12
Piciformes	198	3.89
Procellariiformes	3	0.06
Pteroclidiformes	6	0.12
Sphenisciformes	3	0.06
Strigiformes	66	1.3
Trogoniformes	18	0.35
**(c) The imbalanced training set**	690	
Accipitriformes	24	3.48
Apodiformes	48	6.96
Bucerotiformes	6	0.87
Caprimulgiformes	6	0.87
Charadriiformes	33	4.78
Ciconiiformes	3	0.43
Coliiformes	3	0.43
Columbiformes	15	2.17
Coraciiformes	12	1.74
Cuculiformes	6	0.87
Falconiformes	6	0.87
Galliformes	3	0.43
Gruiformes	15	2.17
Musophagiformes	3	0.43
Otidiformes	3	0.43
Passeriformes	459	66.52
Pelecaniformes	3	0.43
Piciformes	24	3.48
Procellariiformes	3	0.43
Pteroclidiformes	3	0.43
Sphenisciformes	3	0.43
Strigiformes	6	0.87
Trogoniformes	3	0.43
**(d) The imbalanced test set**	345	
Accipitriformes	12	3.48
Apodiformes	24	6.96
Bucerotiformes	3	0.87

Caprimulgiformes	3	0.87
Charadriiformes	15	4.35
Ciconiiformes	3	0.87
Coliiformes	3	0.87
Columbiformes	6	1.74
Coraciiformes	6	1.74
Cuculiformes	3	0.87
Falconiformes	3	0.87
Galliformes	3	0.87
Gruiformes	6	1.74
Musophagiformes	3	0.87
Otidiformes	3	0.87
Passeriformes	219	63.48
Pelecaniformes	3	0.87
Piciformes	12	3.48
Procellariiformes	3	0.87
Pteroclidiformes	3	0.87
Sphenisciformes	3	0.87
Strigiformes	3	0.87
Trogoniformes	3	0.87
