# Supplementary material for: Using pose estimation to identify regions and points on natural history specimens
Source: PLoS Comput Biol. 2023 Feb 22;19(2):e1010933. doi: 10.1371/journal.pcbi.1010933 (PMC9987800; doi:10.1371/journal.pcbi.1010933)
Supplement: S2 Table — After accounting for occluded views of some body regions, the sample sizes across labelled points were: N(reflectance standards 1–5) = 5094, N(Throat, Breast, Belly, Flight feathers) = 1698, N(Mantle) = 1697, N(Coverts) = 1696, N(Crown, Nape) = 1695, N(Tail) = 1678 and N(Rump) = 1422. (PDF) [file pcbi.1010933.s009.pdf]

S2 Table. **Tables of evaluation results for all and individual points for the avian specimen dataset.** After accounting for occluded views of some body regions, the sample sizes across labelled points were: N(reflectance standards 1-5)=5094, N(Throat, Breast, Belly, Flight feathers)=1698, N(Mantle)=1697, N(Coverts)=1696, N(Crown, Nape)=1695, N(Tail)=1678 and N(Rump)=1422.

	**Pixel distance**	**PCK-100**	**Colour correlation coefficients (Bbox-20)**	**Colour correlation coefficients (Heatmap-90)**
**Overall (N=42145)**	47.3	89.3		
**Standards (N=25470)**	21.9	100		
**Standard 1 (N=5094)**	18.2	100		
**Standard 2 (N=5094)**	18.7	100		
**Standard 3 (N=5094)**	20.4	100		
**Standard 4 (N=5094)**	23.9	100		
**Standard 5 (N=5094)**	28.4	100		
**Body Regions (N=16675)**	86.1	73.0	0.914	0.941
**Crown (N=1695)**	42.7	95.0	0.903	0.944
**Nape (N=1695)**	56.4	89.1	0.903	0.93
**Mantle (N=1697)**	77.5	74.8	0.892	0.93
**Rump (N=1422)**	147.4	44.6	0.797	0.834
**Tail (N=1678)**	106.7	65.0	0.802	0.835
**Throat (N=1698)**	52.1	91.2	0.935	0.957
**Breast (N=1698)**	67.8	81.5	0.921	0.948
**Belly (N=1698)**	85.6	72.2	0.937	0.955
**Coverts (N=1696)**	111.5	57.8	0.782	0.853
**Flight feathers (N=1698)**	123.0	54.2	0.798	0.857
