# Supplementary material for: Using pose estimation to identify regions and points on natural history specimens
Source: PLoS Comput Biol. 2023 Feb 22;19(2):e1010933. doi: 10.1371/journal.pcbi.1010933 (PMC9987800; doi:10.1371/journal.pcbi.1010933)
Supplement: S3 Table — These results only include the predictions on bird body regions. We used two-tailed t-test. T-test results with p-values less than 0.05 are in bold text. N is the number of predicted body regions used in the t-tests. (PDF) [file pcbi.1010933.s010.pdf]

S3 Table. Tables of the t-test results comparing pixel distances between predictions using the balanced training set and the imbalanced training set. These results only include the predictions on bird body regions. We used two-tailed t-test. T-test results with p-values less than 0.05 are in bold text. N is the number of predicted body regions used in the t-tests.

	Using the balanced training set		Using the imbalanced training set		T-test results
**The balanced test set**	**Mean pixel distance**	**% of image height**	**Mean pixel distance**	**% of image height**	
Overall (N = 2296)	134.93	4.11	172.61	5.26	**t(2279.5) = -5.12; p = 3.35e-07**
Accipitriformes (N = 100)	133.87	4.08	140.71	4.29	t(97.5) = -0.26; p = 0.799
Apodiformes (N = 100)	111.48	3.40	92.2	2.81	t(85.1) = 1.04; p = 0.3
Bucerotiformes (N = 100)	139.93	4.27	289.11	8.81	**t(59.8) = -2.91; p = 0.00511**
Caprimulgiformes (N = 100)	281.13	8.57	267.66	8.16	t(97.1) = 0.22; p = 0.828
Charadriiformes (N = 100)	154.11	4.70	174.21	5.31	t(97.6) = -0.81; p = 0.422
Ciconiiformes (N = 98)	179.7	5.48	210.47	6.42	t(94.2) = -1.03; p = 0.305
Coliiformes (N = 100)	58.77	1.79	106.78	3.26	**t(64.5) = -2.89; p = 0.00529**
Columbiformes (N = 100)	93.48	2.85	123.81	3.77	t(90) = -1.6; p = 0.113
Coraciiformes (N = 100)	85.56	2.61	115.13	3.51	t(76.6) = -1.49; p = 0.14
Cuculiformes (N = 100)	144.84	4.42	141.04	4.30	t(91.5) = 0.14; p = 0.888
Falconiformes (N = 100)	149.05	4.54	162.08	4.94	t(97.4) = -0.46; p = 0.648
Galliformes (N = 100)	93.81	2.86	326.11	9.94	**t(61.2) = -4.67; p = 1.71e-05**
Gruiformes (N = 100)	151.09	4.61	187.43	5.71	t(95.6) = -0.94; p = 0.348
Musophagiformes (N = 100)	97.43	2.97	132.38	4.04	t(97.9) = -1.46; p = 0.147
Otidiformes (N = 100)	125.89	3.84	190.26	5.80	**t(78.4) = -2.37; p = 0.02**
Passeriformes (N = 100)	92.84	2.83	74.08	2.26	t(93.9) = 1.69; p = 0.0952
Pelecaniformes (N = 98)	201.09	6.13	225.82	6.88	t(96) = -0.68; p = 0.5

Piciformes (N = 100)	105.65	3.22	120.97	3.69	t(97.8) = -0.95; p = 0.342
Procellariiformes (N = 100)	226.93	6.92	201.29	6.14	t(59.5) = 0.37; p = 0.713
Pteroclidiformes (N = 100)	129.46	3.95	170.6	5.20	t(95.2) = -1.43; p = 0.156
Sphenisciformes (N = 100)	107.94	3.29	204.14	6.22	**t(78) = -3.03; p = 0.00336**
Strigiformes (N = 100)	158.4	4.83	204.29	6.23	t(92.3) = -1.76; p = 0.0823
Trogoniformes (N = 100)	83.06	2.53	111.38	3.40	t(76.1) = -1.57; p = 0.12
**The imbalanced test set**					
Overall (N = 2264)	107.19	3.27	110.87	3.38	t(2155.9) = -0.72; p = 0.47
Accipitriformes (N = 72)	124.64	3.80	147.96	4.51	t(67.5) = -1.08; p = 0.283
Apodiformes (N = 160)	89.67	2.73	77.98	2.38	t(137.2) = 0.86; p = 0.389
Bucerotiformes (N = 20)	84.14	2.57	879.77	26.82	**t(9.2) = -4.98; p = 0.000727**
Caprimulgiformes (N = 20)	154.57	4.71	126.42	3.85	t(18) = 0.46; p = 0.648
Charadriiformes (N = 100)	143.52	4.38	127.42	3.88	t(93.9) = 0.75; p = 0.457
Ciconiiformes (N = 20)	87.24	2.66	98.5	3.00	t(14.7) = -0.4; p = 0.694
Coliiformes (N = 20)	62.15	1.89	144.2	4.40	t(10.5) = -1.3; p = 0.22
Columbiformes (N = 40)	140.98	4.30	174.37	5.32	t(37.1) = -0.74; p = 0.466
Coraciiformes (N = 40)	125.32	3.82	147.11	4.49	t(37.2) = -0.75; p = 0.458
Cuculiformes (N = 20)	99.19	3.02	133.07	4.06	t(16.6) = -0.72; p = 0.484
Falconiformes (N = 20)	181.17	5.52	194.68	5.94	t(17.8) = -0.15; p = 0.882
Galliformes (N = 20)	52.43	1.60	270.31	8.24	t(9.1) = -1.68; p = 0.127
Gruiformes (N = 40)	127.18	3.88	131.97	4.02	t(38) = -0.15; p = 0.881
Musophagiformes (N = 20)	67.37	2.05	81.71	2.49	t(17.4) = -1.01; p = 0.325
Otidiformes (N = 20)	105.42	3.21	115.72	3.53	t(14.2) = -0.26; p = 0.795
Passeriformes (N = 1438)	104.87	3.20	89.91	2.74	**t(1354.4) = 2.88; p = 0.00407**
Pelecaniformes (N = 20)	136.02	4.15	174.24	5.31	t(13.4) = -0.99; p = 0.338

Piciformes (N = 76)	104.64	3.19	117.13	3.57	t(74) = -0.55; p = 0.583
Procellariiformes (N = 20)	97.15	2.96	141.65	4.32	t(15.1) = -0.88; p = 0.391
Pteroclidiformes (N = 20)	122.18	3.73	159.45	4.86	t(17.3) = -0.73; p = 0.474
Sphenisciformes (N = 20)	95.59	2.91	161.02	4.91	t(11.1) = -1.31; p = 0.216
Strigiformes (N = 18)	113.4	3.46	140.56	4.29	t(14.7) = -0.96; p = 0.351
Trogoniformes (N = 20)	78.31	2.39	133.61	4.07	t(9.7) = -0.78; p = 0.455

6
