# Supplementary material for: Using pose estimation to identify regions and points on natural history specimens
Source: PLoS Comput Biol. 2023 Feb 22;19(2):e1010933. doi: 10.1371/journal.pcbi.1010933 (PMC9987800; doi:10.1371/journal.pcbi.1010933)
Supplement: S4 Table — We compared the pixel distances between using the balanced training set (690 images), and the leave-one-order-out balanced training set (660 images) on the predictions (15 images per order) from the missing training orders. We used one-tailed t-test, assuming predictions from the balanced training have less pixel distance than the predictions from the leave-one-order-out balanced training set. T-test results with p-values less than 0.05 are in bold text. N is the number of predicted body regions used in the t-tests. (PDF) [file pcbi.1010933.s011.pdf]

S4 Table. **A table of the t-test results on leave-one-order-out tests for each missing order.** We compared the pixel distances between using the balanced training set (690 images), and the leave-one-order-out balanced training set (660 images) on the predictions (15 images per order) from the missing training orders. We used one-tailed t-test, assuming predictions from the balanced training have less pixel distance than the predictions from the leave-one-order-out balanced training set. T-test results with p-values less than 0.05 are in bold text. N is the number of predicted body regions used in the t-tests.

	**Using the balanced training set**		**Using the leave-one-order-out training set**		
**Missing training order**	Mean pixel distance	% of image height	Mean pixel distance	% of image height	**T-test results**
Accipitriformes (N = 100)	133.87	4.08	146.55	4.47	t(97.9) = -0.47; p = 0.321
Apodiformes (N = 100)	111.48	3.4	118.32	3.61	t(97.5) = -0.32; p = 0.373
Bucerotiformes (N = 100)	139.93	4.27	165.82	5.06	t(76.2) = -0.77; p = 0.222
Caprimulgiformes (N = 100)	281.13	8.57	324.02	9.88	t(88.6) = -0.54; p = 0.294
Charadriiformes (N = 100)	154.11	4.7	143.6	4.38	t(98) = 0.41; p = 0.657
Ciconiiformes (N = 98)	179.7	5.48	176.09	5.37	t(95.8) = 0.13; p = 0.55
Coliiformes (N = 100)	58.77	1.79	102.79	3.13	**t(79.4) = -3.59; p = 0.000283**
Columbiformes (N = 100)	93.48	2.85	98.14	2.99	t(93.4) = -0.26; p = 0.398
Coraciiformes (N = 100)	85.56	2.61	104.47	3.19	t(84.6) = -1.08; p = 0.142
Cuculiformes (N = 100)	144.84	4.42	145.67	4.44	t(96.6) = -0.03; p = 0.488
Falconiformes (N = 100)	149.05	4.54	117.61	3.59	t(97.8) = 1.18; p = 0.879
Galliformes (N = 100)	93.81	2.86	350.52	10.69	**t(56.1) = -4; p = 9.51e-05**
Gruiformes (N = 100)	151.09	4.61	143.65	4.38	t(94.7) = 0.23; p = 0.59
Musophagiformes (N = 100)	97.43	2.97	108.19	3.3	t(97) = -0.43; p = 0.333
Otidiformes (N = 100)	125.89	3.84	122.7	3.74	t(96.6) = 0.16; p = 0.562
Passeriformes (N = 100)	92.84	2.83	96.79	2.95	t(97.2) = -0.31; p = 0.379
Pelecaniformes (N = 98)	201.09	6.13	202.66	6.18	t(95.9) = -0.04; p = 0.483

Piciformes (N = 100)	105.65	3.22	123.48	3.76	t(78.8) = -0.81; p = 0.211
Procellariiformes (N = 100)	226.93	6.92	175.37	5.35	t(56.9) = 0.75; p = 0.773
Pteroclidiformes (N = 100)	129.46	3.95	151.33	4.61	t(97.3) = -0.8; p = 0.213
Sphenisciformes (N = 100)	107.94	3.29	123.15	3.75	t(93.1) = -0.6; p = 0.276
Strigiformes (N = 100)	158.4	4.83	171.67	5.23	t(93.9) = -0.52; p = 0.302
Trogoniformes (N = 100)	83.06	2.53	102.85	3.14	t(74.2) = -1.06; p = 0.146

9
