# Supplementary material for: Using pose estimation to identify regions and points on natural history specimens
Source: PLoS Comput Biol. 2023 Feb 22;19(2):e1010933. doi: 10.1371/journal.pcbi.1010933 (PMC9987800; doi:10.1371/journal.pcbi.1010933)
Supplement: S5 Table — The tested networks are Stacked Hourglass and CPM. The tested input resolutions are 494 x 328, 329 x 218 and 247 x 164 pixels. (PDF) [file pcbi.1010933.s012.pdf]

S5 Table. **ANOVA results on pixel distances using different network architectures and input resolutions for the avian specimen dataset.** The tested networks are Stacked Hourglass and CPM. The tested input resolutions are 494 x 328, 329 x 218 and 247 x 164 pixels.

	**F**	**df_1**	**df_2**	**P<0.01**
**Overall**	3529.6	5	252864	TRUE
**Standard 1**	1093.3	5	30558	TRUE
**Standard 2**	2263.7	5	30558	TRUE
**Standard 3**	1368.7	5	30558	TRUE
**Standard 4**	1242.7	5	30558	TRUE
**Standard 5**	1142.0	5	30558	TRUE
**Crown**	154.0	5	10164	TRUE
**Nape**	189.3	5	10164	TRUE
**Mantle**	79.9	5	10176	TRUE
**Rump**	13.2	5	8526	TRUE
**Tail**	81.2	5	10062	TRUE
**Throat**	174.4	5	10182	TRUE
**Breast**	136.2	5	10182	TRUE
**Belly**	66.8	5	10182	TRUE
**Coverts**	56.4	5	10170	TRUE
**Feathers**	49.7	5	10182	TRUE
