# Supplementary material for: Using pose estimation to identify regions and points on natural history specimens
Source: PLoS Comput Biol. 2023 Feb 22;19(2):e1010933. doi: 10.1371/journal.pcbi.1010933 (PMC9987800; doi:10.1371/journal.pcbi.1010933)
Supplement: S6 Table — The four low-quality datasets are: (i) rotation (angles between -45° to 45°), (ii) translation on both x and y axes (-500 to 500 pixels), (iii) horizontal flip 50% images randomly, (iv) the combination of all three transformations. (PDF) [file pcbi.1010933.s013.pdf]

S6 Table. **ANOVA results on pixel distances of overall and individual points across the original and four tested low-quality avian specimen datasets.** The four low-quality datasets are: (i) rotation (angles between -45° to 45°), (ii) translation on both x and y axes (-500 to 500 pixels), (iii) horizontal flip 50% images randomly, (iv) the combination of all three transformations.

	**F**	**df_1**	**df_2**	**P<0.01**
**Overall**	87.2	4	210720	TRUE
**Standard 1**	532.7	4	25465	TRUE
**Standard 2**	260.0	4	25465	TRUE
**Standard 3**	146.1	4	25465	TRUE
**Standard 4**	33.5	4	25465	TRUE
**Standard 5**	18.4	4	25465	TRUE
**Crown**	9.9	4	8470	TRUE
**Nape**	10.1	4	8470	TRUE
**Mantle**	7.3	4	8480	TRUE
**Rump**	1.7	4	7105	FALSE
**Tail**	9.1	4	8385	TRUE
**Throat**	8.4	4	8485	TRUE
**Breast**	6.7	4	8485	TRUE
**Belly**	3.3	4	8485	TRUE
**Coverts**	12.6	4	8475	TRUE
**Feathers**	11.8	4	8485	TRUE
