# Supplementary material for: Using pose estimation to identify regions and points on natural history specimens
Source: PLoS Comput Biol. 2023 Feb 22;19(2):e1010933. doi: 10.1371/journal.pcbi.1010933 (PMC9987800; doi:10.1371/journal.pcbi.1010933)
Supplement: S7 Table — (PDF) [file pcbi.1010933.s014.pdf]

S7 Table. **MANOVA results on the effect of ecotypes and labelling methods (i.e. experts or the Stacked Hourglass) on shape variation (PC1-8).**

	DF	Approx. F	DF1	DF2	P
**Ecotype**	1	124.49	8	367	<0.0001
**Labeller**	1	0.35	8	367	0.95
