# Supplementary material for: Using pose estimation to identify regions and points on natural history specimens
Source: PLoS Comput Biol. 2023 Feb 22;19(2):e1010933. doi: 10.1371/journal.pcbi.1010933 (PMC9987800; doi:10.1371/journal.pcbi.1010933)
Supplement: S8 Table — (PDF) [file pcbi.1010933.s015.pdf]

S8 Table. Group distances among the combinations of ecotypes and labelling methods.

Group 1	Group 2	Group Distance
Crab & Ground Truth	Crab & Deep Learning	0.00351
Crab & Ground Truth	Wave & Ground Truth	0.05727
Crab & Ground Truth	Wave & Deep Learning	0.05469
Wave & Ground Truth	Wave & Deep Learning	0.00865
Wave & Ground Truth	Crab & Deep Learning	0.05729
Wave & Deep Learning	Crab & Deep Learning	0.05508
