# Supplementary material for: Using pose estimation to identify regions and points on natural history specimens
Source: PLoS Comput Biol. 2023 Feb 22;19(2):e1010933. doi: 10.1371/journal.pcbi.1010933 (PMC9987800; doi:10.1371/journal.pcbi.1010933)
Supplement: Attachment — Submitted filename: response_to_reivewers.pdf [file pcbi.1010933.s017.pdf]

Editor:

I agree with both reviewers that this is a very interesting study and method that potentially has high value. However, I share reviewer 2's concern about the representativeness of the training data. I could envision three possible responses:

1.) Perform a series of cross-validation experiments where the data set is split such that the training data has equal representation across the taxa. You could also perform leave-one-out cross validation where each taxon is left out completely from the training data set. These results would pretty clearly demonstrate whether the data bias matters.

First, we would like to thank the editor for their comments. To address the issue of imbalance across taxa, we labelled a new *training* set with a balanced number of images per order (30 images per order for 23 orders with >45 images, 690 images total) and also labelled a balanced *test* set (15 images per order for the same 23 orders, 345 images total) that have equal representation across the orders. We also sampled an imbalanced training set (690 images, 30 images per order for 23 orders) and an imbalanced test set (345 images, 15 images per order for 23 orders), trying to make them have similar imbalanced distribution to the full dataset. We used the balanced and imbalanced training sets to train different models and then applied them to the balanced and imbalanced test sets. We also applied leave-one-order-out on the balanced training set and used these leave-one-order-out sets as the training sets.

These analyses showed that the balanced training set performed well on both the balanced and imbalanced test sets, the imbalanced training set only performed well on the imbalanced test set. This result suggests that having a training set that is representative of the full dataset (either balanced or imbalanced) would be a good practice. In addition, lacking images from a certain taxonomical group in the training set would limit the Deep Learning performance of that group. We have added these new analyses and results to the manuscript (lines in the tracked manuscript: 393-409; 555-590; 712-730; Fig. 7).

2.) Secure additional data to more evenly balance the taxonomic representation in the training data. Obviously this would require a substantial amount of work and may functionally be impossible. I only suggest this on the off chance it's more straightforward for the authors than it seems.

It is not possible to generate additional data to balance out taxonomic representation as some bird orders simply contain very few species whereas others are more species rich. Therefore, as mentioned above in our response to comment (1), to address the issue of imbalance we created a balanced training set and test set by downsampling our original dataset and compared model performance based on imbalanced and balanced datasets.

3.) Carefully re-frame the manuscript to focus on the methods, as opposed to any interpretation from a biological perspective and/or clearly state that the generalizability of the method is left for future work.

We assume the biological parts the editor pointed out might be (1) the interpretation of error rates from a biological perspective given the variation in sample size among orders, (2) the morphospaces from both Deep Learning and manual landmarks or (3) the plumage colour measured from body region points.

For (1), we agree it is inappropriate to interpret error rates from a biological perspective given the variation in sample size among orders. So, we have removed this part (lines in

the tracked manuscript: 361-364; 530-535; Old S2 Table) and hope that this helps to address this concern.

For (2) and (3), the main reason we included these two parts is: we want to measure if morphospaces or plumage colours from Deep Learning and manual labelling are similar, which suggests that Deep Learning predictions can generate biologically meaningful measurements. In the discussion, we discussed that morphospaces and colour correlation are complementary metrics to direct metrics (i.e. pixel distance and PCK), as they show the biologists that the predictions are accurate from a biological perspective. We hope the editor can understand why we included part (2) and (3), but we are also happy to move them to supplementary material if required. Discussions on (2) and (3) were added. (lines in the tracked manuscript: 678-683)

My advice would be to take a hybrid approach between suggestions 1 and 3, but I will of course leave that to the discretion of the authors.

We thank the editor for this constructive advice. As detailed above, to address the issue of representativeness in our original training set, we have followed suggestions 1 and 3.

Reviewer #1

First, I would like to thank the author for their work. Please if any of my comment sounds out of subject because it is due to my misunderstanding, I apologize in advance. Thus it also mean that this specific parts where I misunderstood maybe require some clarification.

This interesting study aims at using deep learning models and more specifically pose estimation to identify body regions and colour of these regions in birds as well as points on snail shells. The manuscript consider multiple network architecture as well as input to identify the best parameters to train such models. The details in the “Model evaluation” section were appreciated. Each evaluation criteria was justified.

We would like to thank the reviewer for the comments on our manuscript. The comments are very helpful. We have updated the manuscript based on these comments and we think the revised version is much improved.

Minor issue

Line 167. “The sample of 1698 bird species includes representatives of 81% of bird genera and 27 bird orders, so the labelled images should capture much of the extent of variation in plumage colour, patterns, and body shape among birds.”. However, one important information for deep learning training is if there is some class imbalance in the training dataset. S2 Table show as the “N” per class, that there is a high imbalance between classes (come classes represented with 3 pictures and other up to 3405 pictures. And this can definitely impacted the network performances.

We apologise for the lack of clarity regarding sample sizes in our original manuscript. In the revised version, we clearly state the reasons why the 5094 genus-level dataset is imbalanced across orders. Specifically, we now describe the full dataset (234,954 images), from which the 5094 genus-level dataset is sampled from. This is done to indicate that the full dataset is taxonomically imbalanced as the number of images (species sampled) per order simply follows the pattern of species richness imbalance across orders inherent in the bird phylogeny (e.g. the order Passeriformes contains more than 60% of the species in the bird tree). We have rewritten the relevant section to give

more detail about the underlying sampling structure of the full dataset (lines in the tracked manuscript: 150-209).

Nonetheless, as mentioned above, to address the imbalance issue we additionally created a balanced training set (30 images per order for 23 orders with >45 images, 690 images total) and a balanced test set (15 images per order for the same 23 orders, 345 images total). We also sampled an imbalanced training set (690 images, 30 images per order for 23 orders) and an imbalanced test set (345 images, 15 images per order for 23 orders), trying to make them have similar imbalanced distribution to the full dataset. We used the balanced and imbalanced training sets to train different models and then applied them to the balanced and imbalanced test sets. We also tried leave-one-order-out on the balanced training sets, creating 23 training sets without a certain order. We used each of them to train a model. By doing these tests, we can evaluate the impact of imbalanced data on Deep Learning performance.

These analyses showed that the balanced training set performed well on both the balanced and imbalanced test sets, the imbalanced training set only performed well on the imbalanced test set. This result suggests that having a training set that is representative of the full dataset (either balanced or imbalanced) would be a good practice. In addition, lacking images from a certain taxonomical group in the training set would limit the Deep Learning performance of that group. As mentioned above, we have added these new analyses and results to the manuscript (lines in the tracked manuscript: 393-409; 555-590; 712-730; Fig. 7).

Linked with the previous comment, the objective to test “the relationship between bird taxonomy and network performance to assess whether performance varies among groups of bird species due to broad differences in size, shape and colouration of specimens” (Line 231) is directly impacted by class imbalance. Indeed, if the in the training data set there are birds’ orders that are quite singular and rarely represented (for example the “Sphenisciformes” N=3), then the model will simply trained and adapt its weights only 3 times per epoch to try to fit to this order. So in order to access whether the performance varies among groups of bird species, I would suggest to either increase the dataset to have approximately the same number of pictures per birds’ orders (but I understand that this may be a lot of work). Alternatively, I would suggest to implement some class weights in the model or to use data augmentation that would still keep the standardize point of view (cropping, zooming, slightly blurring the picture, ...).

As mentioned in the previous response, we now also use a balanced training set and a balanced test set to see how dataset balance effect model performance. We mentioned image augmentation in the discussion as a possible solution for underrepresented groups.

We also agreed that presenting error rates per order for an imbalanced dataset in our previous draft may be misleading, we therefore removed this part (lines in the tracked manuscript: 361-364; 530-535; Old S2 Table)

Line 228 “We used only the training and validation sets so that every image from the labelled dataset can have a predicted point from the same data partition routine.” Here the justification of why not using a test set was not very clear. However, I have some concern about using only training and validation sets with the cross validation. For what I understand in the text, the 5094 pictures were used for the cross-validation (all the run having 80% training and 20% validation). If it is not the case and that the 20% of

validation set were never seen by the model during the training phase then it's alright. However, if all the pictures were used during the training, then when evaluation the model what is evaluated is the capacity of the neural network to learnt "by heart" the features on this pictures, but not how to recognize the features on pictures. As an example, if you train a student with an exercise where the solution is given at the end, if when evaluating the student you give the exact same exercise, then only the capacity to remember the exercise will be evaluating, not his capacity to understand how to fix the exercise. As a researcher, I would be interested to know if this model as it is right now, would work on a species that was not used during training. Thus I would nevertheless suggest to have a small test dataset (200-300 pictures to access the capacity of the model to detect regions and colours of totally new pictures (either same species and new individuals or even new species).

To answer the reviewer's question ("If it is not the case and that the 20% of validation set were never seen by the model during the training phase then it's alright"): the validation images were not seen by the model during training, thus alleviating the reviewer's primary concern. We also made changes to the part of creating the training set and the validation set to clarify how the dataset is split (lines in the tracked manuscript: 261-278).

We add a balanced test set, which is a new dataset that is completely different from the 5094 genus-level dataset. The new test set has 345 new images, and it was used to evaluate two models (trained by either the balanced or the imbalanced training set), the model using the balanced training set outperformed the one using the imbalanced training set.

To address the reviewer's comment "I would be interested to know if this model as it is right now, would work on a species that was not used during training.", we tried leave-one-order-out on the balanced training sets. Only 2 out of 23 orders were significantly worse when not having images from these orders in the training set.

To address the reviewer's comment on creating a small test dataset (200-300 pictures), we test the model on the balanced test set (345 images) mentioned in the previous response, which is newly selected from the full dataset (i.e. not from the 5094 genus-level dataset).

This may also explain why "the model does not predict labels from non-trainers as reliably" (Line 474). Indeed, if the models was trained using one dataset, it has learn by heart how to deal with these pictures and since the training was done using the trainer annotation, it would be indeed quite close from the trainers' annotation. To test the differences between predictions, trainer annotations and non-trainer annotation, I would suggest to use the test dataset mentioned previously as a completely independent dataset.

By comparing predictions to trainer annotations and non-trainer annotations, we aim to show that the difference between prediction and trainer is similar to the difference between the trainer and non-trainer. This implies that Deep Learning predicted body regions of birds are of comparable accuracy to predictions from non-trainer human annotators. Also, we want to show that Deep learning prediction is closer to the trainer's than non-trainers'. To test these differences, we used the predictions from the 5094 genus-level datasets. The predictions are the outputs that the model predicted on the validation set (images that are not seen by the model during training. See lines in the

tracked manuscript: 261-278), so they are from an independent set. We think this analysis would work on the predictions sampled from the 5094 genus-level dataset. We hope the reviewer can understand why we use images from the 5094 genus-level dataset.

All of this do not make incorrect the main conclusion of deep learning being able to detect regions on pictures. The great novelties that this study brings, are it's application for biological questions and the use of large museums datasets. The previous comments are just here to balance some of the results and discussion.

Again, we thank the reviewer for giving us very constructive comments.

Line 514-521. The results are presented using PC axis, but I would have appreciated to maybe have a biological results as well. Such as which one of the landmarks would account for most of the variability in snail shell shape between the different ecosystems. However, this do not really make the paper not understandable.

As mentioned above, we have carefully considered the level of biological results that should be included in this manuscript, and we decided not to add more biological results. The main reason is introducing biological analysis might distract from the focus of this study. In the paper, we showed readers that biological measures (i.e. plumage colour and the morphospace) from Deep Learning are accurate when compared with manual labelling. We think these results were sufficient for biologists to know Deep Learning predictions can generate biologically meaningful measurements. new discussions were added (lines in the tracked manuscript: 678-683).

We did not show which landmark would account for the most variability in the shape, as it could introduce more biological analysis and potentially unbalance the manuscript. However, for additional insight into patterns of variability among landmarks, we have updated Fig.8 (Fig.7 in the previous version), adding visualisations of shell shapes and landmarks along the PC 1 and 2 axes.

Line 569. One of the discussion of the paper it to encourage researchers to "manually checking the predictions". I have the feeling that this is in contradiction with the main argument for using deep learning as set in the introduction. The objective was to create a tool that will help gain time. I do not say that the predictions are not to be checked, but checking manually thousands of predictions (if the dataset is large) can be quite time consuming. The all point of evaluating the model is to evaluate if the model can be trusted regardless a certain amount of errors. If the overall accuracy of the model is not enough then, the model should not be trusted and would need more fine tuning. However if the model is accurate enough, then maybe using a threshold on the confidence score would be a first step to select the potential wrong predictions that would need to be manually checked (but it would be a small subset of the whole dataset).

We added more discussion on how to post-process the predictions to increase the accuracy. We discussed the possible solutions of using statistical tests to flag some possible errors and review images that have a higher chance of being incorrectly predicted. By using these solutions, the review process can be more efficient (lines in the tracked manuscript: 684-697).

Comments:

Line 163. Are the non-labelled regions still detected somehow by the neural network ? I wonder if the network, after trained to detect different body regions on animal with variation in plumage colour, patterns and body shape, can outperformed humans and detect regions that are difficult for humans to annotate (This is purely a personal interest and I am not expecting for this to be updated in the manuscript).

Yes, it will detect the non-labelled regions as well. For example, if a rump is covered by wings, the rump prediction would normally be placed on the location where wings cover the rump. However, this situation only occurs rarely, less than 2% of the body regions were not labelled in the 5094 genus images.

Line 763. S2 Fig. No (a) and (b) on the figure but this is just a small thing to adjust.

We have updated Fig. S2. The new Fig. S2 now only contains the previous panel (a) as we removed the result displayed in (b) following comments from Reviewer 2.

Reviewer #2:

General comment:

This work proposes a deep learning-based approach to automatically identify body regions from digitised biological specimens, namely birds and Littorina. I think this works makes a significant contribution to the field and has the potential to be very useful for researchers working with large datasets. The manuscript is very well organized and the authors are clear in most of their methodology, which is relatively easy to follow by others. However, I think that there is some room for improvement and some points should be clarified. I make my comments per section below:

We would like to thank the reviewer for their constructive comments. We have updated the manuscript based on these comments.

<u>Introduction</u>

The introduction is very well written and introduces well the scope of the work.

<u>Methods</u>

159: It would be nice to present the average number of images per bird species and the minimum and maximum number of pictures to give a better idea of the dataset or reference directly to table S2.

We have added a new table (Table S1 in the revised version) which presents the image count and proportion per order of the datasets we introduced in the data section.

224-233: I think that the justification for not using a test dataset is not well supported, especially because the authors claim to have 245,220 images (line 596). This is more than enough data to create an additional dataset potentially containing different species that were not included in the training data. This would give information on the general application of the trained model and an independent evaluation measurement. Furthermore, I think that having orders with very few images is a major issue in this work that is not properly addressed. Having a test dataset with a balance sampling scheme, for example 50 images for each order, would give a better idea of the deep learning model's performance and how it varies with the number of samples in the training dataset. On table S2 the authors provide an idea of the error rate per order, but this is

done with a very variable number of pictures per order (for example some orders have only 3 images to train and determine the error rate). If you want to provide guidelines on how to build a deep learning-based algorithm for any task, the question what sample size is needed? Is probably the first question that researchers ask and is not answered in this work.

We would like to apologise that we did not give enough detail about our datasets. In the revised version, we introduced the datasets (the full dataset, the 5094 genus dataset, the balanced training set and the balanced test set) in the data section (lines in the tracked manuscript: 150-209). We also corrected the number of images for the full dataset (234,954 instead of 245,220). We have also rewritten the d) Model Training section (lines in the tracked manuscript: 261-278), where we now give more detail on how the training set (80% of the 5094 genus-level dataset) and the validation set (20% of the 5094 genus-level dataset) is split, and clarified that the validation images were not used during the model training.

As outlined above, to address comments regarding dataset imbalance we added a balanced training set (690 images, 30 images per order for 23 orders) and a balanced test set (345 images, 15 images per order for 23 orders). We also sampled an imbalanced training set (690 images, 30 images per order for 23 orders) and an imbalanced test set (345 images, 15 images per order for 23 orders), trying to make them have similar imbalanced distribution to the full dataset. We used the balanced and imbalanced training sets to train different models and then applied them to the balanced and imbalanced test sets.

Regarding error rates, we agree with the reviewer's comments that presenting error rates per order for an imbalanced dataset may be misleading. Therefore, as mentioned, we have removed the previous version of Table S2 containing this information. Instead of examining error rates per order, we now compare the performance (overall and per order) between models trained using the balanced training set and the imbalanced training set.

These new results showed that the balanced training set performed well on both the balanced and imbalanced test sets, the imbalanced training set only performed well on the imbalanced test set. This result suggests that having a training set that is representative of the full dataset (either balanced or imbalanced) would be a good practice. As mentioned above, these new analyses and results have been added to the manuscript (lines in the tracked manuscript: 393-409; 555-590; 712-730; Fig. 7).

260-263: Does this point here implies a manual post-processing task? Wouldn't it be possible to automatically determine if some of the body parts are not present? For example, the model could output an empty heatmap for a given body part when not present, or could you add as additional inputs a variable for each body part with 1 for present and 0 for not present. Or even having an additional Deep learning model to determine which body parts were present. How would your system work when analysing thousands of pictures for which there is no information on how many body parts are occluded?

We tried to run a Deep Learning model to classify if a point is occluded (not visible) in an image. However, the predictions did not predict occluded points well, possibly due to the number of occluded points being very small (less than 2% of points are occluded in the 5094 genus images) and the model might not be effectively trained. Because the occluded points are very few and we want to focus on predicting the points, we do not

compare the body region points if the corresponding ground truth point is occluded. Therefore the post-process of finding the occluded points (only for the labelled dataset) in the paper is automatic. Further works need to be done if we want to automatically detect the occluded points for the rest of the images (without manual labels).

313: It needs to be clearer how did you cross the predictions with the manually boundaries of the body parts. For example, did you use any software to draw polygon lines delimiting each body part and then estimated if any point of the prediction was outside this polygon region?

We clarified how we identify the body regions, where experts visualise the regions virtually on images (lines in the tracked manuscript: 358-359).

Results

445-453 Although the correlations are high it would be important to understand if this is similar across taxa or if this is a result of some orders being overrepresented. As it is currently present it is not clear if your model works for all the 27 orders or for Passeriformes, but not for Galliformes, for example.

We showed the pixel distances (overall and per order) on the balanced test sets (23 orders used) in S3 Table, so readers can see the model performance on each order. And we showed that using a balanced training set outperformed using an imbalanced training set on the balanced test set.

458- I think that the reasons for an image to be considered incorrect should have been defined *a prior*, if you want to conclude something about this. For example, before crossing the data, it would have better to classify features of the pictures *a priori*, such as: “far”, “close”, “low quality”, “obscure” etc… Or more simply: “hard” vs “easy” for manual labelling. And then actually test if for example, “hard” pictures were more likely to be considered as incorrect. Otherwise this result sounds very subjective.

The reviewer’s comment about using a prior for the image difficulties is very useful to show the results objectively. We went through the images (just the images without knowing any results) and we classified the images based on the difficulties from a human’s perspective. We then compared the predictions to this prior. The result section and supplementary material were updated based on this comment (lines in the tracked manuscript: 523-529).

Discussion

587- Table 2 tries to give some recommendations about building a Deep learning Pipeline. Although I think this a good idea, I think that some points need to be clearer:

Regarding digitisation I would argue that including images of different setups would actually contribute to a better generalization of the deep learning model. If you train a model only with your setup it is more likely that the model will perform badly when other researchers use your model on their own setups.

For a new project, we would recommend training the model with a training set from the full dataset (point 3 in Table 2). Then applying the trained model to the rest of the data. A project consisting of highly standardised images would have a better result. In point 1, we also mentioned that acceptable results can be gained even with less consistent images. Evidence for this comes from our work: we ran the model on low-quality datasets

and the performance did not markedly decrease, indicating that Deep Learning models are very robust on less consistent datasets.

The second point on digitisation needs to be supported either with previous related work or with data. You could, for example, use your trained model to make predictions in a dataset that was collected with a different setup, or at a different museum. If you can do that, your statement will be more credible.

As mentioned in our last response, we ran the model on several low-quality datasets where we randomly moved rotated, flipped, and zoomed the original images. The accuracies for the low-quality datasets were worse than for the original dataset, but the decreases in accuracy were relatively small (pixel distance smaller than 10 pixels). See lines in the tracked manuscript: 385-392; 600-607; 741-744 and S4 Fig.

In previous studies, the model accurately identified human postures on real-world images, where the backgrounds were more complex. On the other hand, images with higher consistency would make the model easier to learn and make better predictions. Therefore we recommend researchers digitise images in a highly consistent setup if possible, but it’s ok to have less consistent images (point 1 in Table 2).

Regarding labelling it is stated that labelling should be representative of the whole dataset. Nevertheless in your work you have orders with very few samples (<4 images), which are not representative of the whole dataset and there is no discussion about this issue.

Based on the result of analysing taxonomic imbalance (i.e. compared using balanced and imbalanced sets), we suggested researchers create their training sets to be representative of the whole datasets.

Regarding evaluation, I think that it is of critical importance to evaluate the model on a test dataset that contains images that are independent from the training dataset (e.g. images from different individuals, species, setups, collections, museums, photographer, etc…). This would give information on the general application of the trained model. In the case of your specific model, since you did not test it on a different dataset, it is not clear if anyone else could use it directly on their datasets that might have been collected with a different camera quality, include different species, etc…

The pipeline in our manuscript suggests researchers create their own training sets (point 2 in Table 2). Creating specialised training sets will undoubtedly maximise the model’s performance. However, we did perform some analyses to investigate model robustness on images with a less consistent setup. We trained four models on four low-quality datasets generated by randomly applying pan, rotation, zoom and horizontal flip on the 5094 genus dataset (See lines in the tracked manuscript: 385-392). Predictions based on these low-quality training datasets were nonetheless quite accurate (<10 pixels worse than the original dataset), implying that our model may be useful for predicting points on non-identical datasets. However, we agree that the general applicability of applying the model to datasets collected in very different ways remains to be determined.

Regarding post-processing I think that manual checking is feasible for small datasets, but it is a bottleneck if the aim is to apply an automated approach to a large scale datasets. I think that you should consider adding a “flagging” system. For example, if in all pictures the head is always on the left side of the picture and the tail on the right then every time the prediction of the head if predicted to be more to the right than the tail you should flag automatically this image. More criteria could be implemented, for

example, from the manually annotated pictures you could draw a distribution of distances between the body parts for a given order. When analysing the predictions if two body parts are closer or further way from what is usually observed you could flag that prediction for manual checking. This would speed up the post-processing stage.

We added more discussion on how to post-process the predictions to increase the accuracy. We discussed the possible solutions of using statistical tests to flag some possible errors and review images that have a higher chance of being incorrectly predicted. By using these solutions, the review process can be more efficient (lines in the tracked manuscript: 684-697).

Finally, checking for orders for which the error rate is larger needs to be taken with caution. For example, in your model there is 0% error for Eurypygiformes. However, this error rate is estimated from 3 images only. If I use your model on a different dataset with dozens of Eurypygiformes images, should I never manually check this order? This leads me to another missing issue in this table that is how to deal with unbalanced datasets. Should I include an order for which I have 3 samples? Can you confidently claim that you model works on Eurypygiformes with >95% accuracy solely based on 3 images?

We addressed the reviewer's previous comment "224-233: I think that the justification for not using a test dataset is not well supported…" by comparing the performance between using balanced and imbalanced sets (for both training and test sets). We used these results to replace the error rate per order. See detailed responses above.
