# Supplementary material for: Using pose estimation to identify regions and points on natural history specimens
Source: PLoS Comput Biol. 2023 Feb 22;19(2):e1010933. doi: 10.1371/journal.pcbi.1010933 (PMC9987800; doi:10.1371/journal.pcbi.1010933)
Supplement: Attachment — Submitted filename: Review.docx [file pcbi.1010933.s016.docx]

General comment:

This work proposes a deep learning-based approach to automatically identify body regions from digitised biological specimens, namely birds and Littorina. I think this works makes a significant contribution to the field and has the potential to be very useful for researchers working with large datasets. The manuscript is very well organized and the authors are clear in most of their methodology, which is relatively easy to follow by others. However, I think that there is some room for improvement and some points should be clarified. I make my comments per section below:

Introduction

The introduction is very well written and introduces well the scope of the work.

Methods

159: It would be nice to present the average number of images per bird species and the minimum and maximum number of pictures to give a better idea of the dataset or reference directly to table S2.

224-233: I think that the justification for not using a test dataset is not well supported, especially because the authors claim to have 245,220 images (line 596). This is more than enough data to create an additional dataset potentially containing different species that were not included in the training data. This would give information on the general application of the trained model and an independent evaluation measurement. Furthermore, I think that having orders with very few images is a major issue in this work that is not properly addressed. Having a test dataset with a balance sampling scheme, for example 50 images for each order, would give a better idea of the deep learning model’s performance and how it varies with the number of samples in the training dataset. On table S2 the authors provide an idea of the error rate per order, but this is done with a very variable number of pictures per order (for example some orders have only 3 images to train and determine the error rate). If you want to provide guidelines on how to build a deep learning-based algorithm for any task, the question what sample size is needed? Is probably the first question that researchers ask and is not answered in this work.

260-263: Does this point here implies a manual post-processing task? Wouldn’t it be possible to automatically determine if some of the body parts are not present? For example, the model could output an empty heatmap for a given body part when not present, or could you add as additional inputs a variable for each body part with 1 for present and 0 for not present. Or even having an additional Deep learning model to determine which body parts were present. How would your system work when analysing thousands of pictures for which there is no information on how many body parts are occluded?

313: It needs to be clearer how did you cross the predictions with the manually boundaries of the body parts. For example, did you use any software to draw polygon lines delimiting each body part and then estimated if any point of the prediction was outside this polygon region?

Results

445-453 Although the correlations are high it would be important to understand if this is similar across taxa or if this is a result of some orders being overrepresented. As it is currently present it is not clear if your model works for all the the 27 orders or for Passeriformes, but not for Galliformes, for example.

458- I think that the reasons for an image to be considered incorrect should have been defined *a prior*, if you want to conclude something about this. For example, before crossing the data, it would have better to classify features of the pictures *a priori*, such as: “far”, “close”, “low quality”, “obscure” etc… Or more simply: “hard” vs “easy” for manual labelling. And then actually test if for example, “hard” pictures were more likely to be considered as incorrect. Otherwise this result sounds very subjective.

Discussion

587- Table 2 tries to give some recommendations about building a Deep learning Pipeline. Although I think this a good idea, I think that some points need to be clearer:

Regarding digitisation I would argue that including images of different setups would actually contribute to a better generalization of the deep learning model. If you train a model only with your setup it is more likely that the model will perform badly when other researchers use your model on their own setups.

The second point on digitisation needs to be supported either with previous related work or with data. You could, for example, use your trained model to make predictions in a dataset that was collected with a different setup, or at a different museum. If you can do that, your statement will be more credible.

Regarding labelling it is stated that labelling should be representative of the whole dataset. Nevertheless in your work you have orders with very few samples (<4 images), which are not representative of the whole dataset and there is no discussion about this issue.

Regarding evaluation, I think that it is of critical importance to evaluate the model on a test dataset that contains images that are independent from the training dataset (e.g. images from different individuals, species, setups, collections, museums, photographer, etc…). This would give information on the general application of the trained model. In the case of your specific model, since you did not test it on a different dataset, it is not clear if anyone else could use it directly on their datasets that might have been collected with a different camera quality, include different species, etc…

Regarding post-processing I think that manual checking is feasible for small datasets, but it is a bottleneck if the aim is to apply an automated approach to a large scale datasets. I think that you should consider adding a “flagging” system. For example, if in all pictures the head is always on the left side of the picture and the tail on the right then every time the prediction of the head if predicted to be more to the right than the tail you should flag automatically this image. More criteria could be implemented, for example, from the manually annotated pictures you could draw a distribution of distances between the body parts for a given order. When analysing the predictions if two body parts are closer or further way from what is usually observed you could flag that prediction for manual checking. This would speed up the post-processing stage.

Finally, checking for orders for which the error rate is larger needs to be taken with caution. For example, in your model there is 0% error for Eurypygiformes. However, this error rate is estimated from 3 images only. If I use your model on a different dataset with dozens of Eurypygiformes images, should I never manually check this order? This leads me to another missing issue in this table that is how to deal with unbalanced datasets. Should I include an order for which I have 3 samples? Can you confidently claim that you model works on Eurypygiformes with >95% accuracy solely based on 3 images?
