# Supplementary material for: Using pose estimation to identify regions and points on natural history specimens
Source: PLoS Comput Biol. 2023 Feb 22;19(2):e1010933. doi: 10.1371/journal.pcbi.1010933 (PMC9987800; doi:10.1371/journal.pcbi.1010933)
Supplement: Attachment — Submitted filename: response_to_reviewers_v2.docx [file pcbi.1010933.s018.docx]

**Reviewer #1:**

I would like to thank the authors for this revised version of the manuscript and the answers to the comments of the editor and the reviewers. The manuscript presents lot of improvements:

- The method section “Specimen imaging and annotation” is much clearer than before about the composition of the different dataset (the balanced, imbalanced, 5094 genus-level). It is much more understandable about what was done and used to create the models.

- Comparing the effect of balanced vs imbalanced training set on similar balanced and imbalanced testing set is good in my opinion. This will really help to emphasize the importance of taking time to think and create the appropriate training et before running deep learning models.

- The model comparison of the leave-one-order-out is a nice improvement.

We are pleased that the reviewer is happy with our revisions and additional analysis. We would like to thank the reviewer for the comments on our manuscript. We have further updated the manuscript based on these new comments.

However, I still have some comments and questions about few specific parts of the study.

Line 262. Regarding my previous comment: “For what I understand in the text, the 5094 pictures were used for the cross-validation (all the run having 80% training and 20% validation). If it is not the case and that the 20% of validation set were never seen by the model during the training phase, then it’s alright.”. You answered that “the validation images were not seen by the model during training”, but that’s not how the cross validation works and not how you wrote it in the manuscript. You divided the 5094 pictures dataset in 5 batch and each round (there are 5 rounds as you used five-fold cross-validation) you choose one batch as the validation and the rest as the training, and you change every time. Meaning that at the end, all the 5094 pictures are seen by the model to train and to evaluate (line 268 “every image from the 5094 genus-level set can be predicted and evaluated once”). So, all the validation images were seen by the model during training, not at the first cross validation but at the second one or until the fifth round.

I understand that you want to do that way because you only have one specimen per genus and thus you want the model to see all the genus in the dataset.

In the manuscript it is well written but your answer to my comment was different. Therefore, I explained the difference here.

One comment of the previous revision was to create a new dataset with images that the model has never seen. I think that is what you did with the new analysis of balanced vs imbalanced training set, were you have the training and the test that are pictures never used to validate the model at the end of each epoch.

So, for me it is ok to not have a test set for the model using the 5094, but I would suggest to detailed that you do not need it, as you have been testing the imbalanced training set with both balanced and imbalanced testing set.

Line 299. The previous comment leads to “After training, validation images were fed into the trained network to generate prediction heatmaps.”

I understand this sentence and the process for the balanced and imbalanced dataset: training is used to train and the test that are completely new pictures are the validation images in this sentence.

But I did not understand then what are these validation images for the 5094 genus-level model. Are they the 5094 genus-level pictures? but they are the one used for training so performing predictions on the same pictures used to train is not ideal. Or are they other pictures?

We apologise for the lack of clarity regarding the training and validation part, and we updated this part (line 258 – 264 for the highlighted version).

Here we summarise what we did for training and validating 5094 images. For each time (round) we use one batch (from five batches) as the validation set, and we start to train an un-trained model from the beginning (i.e. the model with all new parameters) for 15 epochs. Therefore we have five independently trained models, and validation images were not seen by the Stack Hourglass. We think our approach can be called five-fold cross validation, as it follows the definition of k-fold cross validation (https://en.wikipedia.org/wiki/Cross-validation_(statistics)#k-fold_cross-validation).

Line 720 (and 578). “So using a training set that is representative of the full data could again be an optimal solution for datasets that are either taxonomically balanced or imbalanced.”. I quite disagree with this conclusion. I think that showing this comparison and these results is important. But I do not see how to conclude that it is important to have a training set representative of the full data either balanced of imbalanced. In your answer to the reviewers’ comments, you wrote: “These analyses showed that the balanced training set performed well on both the balanced and imbalanced test sets, the imbalanced training set only performed well on the imbalanced test set.”. Then wouldn’t it be more useful to always use a balanced dataset?

If later one research team, which is focusing on Eurypygiformes for example, wants to use your model to detect the colour of body regions of hundreds of specimens, they may be able to use the model trained using the balanced set, but it would not work if they use the model trained with the imbalanced set.

Therefore, regarding your results, I would rather conclude to always use a balanced dataset for training, to reach good prediction levels on both balanced and imbalanced new dataset to predict.

Based on our results, balanced training sets should be useful for both balanced and imbalanced datasets. However, this is based on both training sets that have the same size. Sometimes creating balanced training sets can make the size small, as there aren’t enough images from underrepresented groups. Using an imbalanced but representative training set can create a larger training set. In the discussion, we suggest using training sets that are representative of the full dataset (either balanced or imbalanced). (Line 689 – 711 for the highlighted version)

For the reviewer’s example of using our model (trained) on Eurypygiformes or other absent groups, we mentioned in the discussion that the model trained on our training set could limit the performance of those groups. And we suggest building a new training set with images from those groups and train the model from the beginning. (Line 712 – 722 for the highlighted version).

**Reviewer #2:**

I appreciate the effort of the authors in clarifying many of the points that were raised and I think that the manuscript improved significantly. I have no major comments and I'm confident that this manuscript will make a significant contribution to make data obtained from biological specimens more accessible.

We are pleased that the reviewer is happy with our revisions and we would like to thank the reviewer for the comments on our manuscript. We have further updated the manuscript based on these new comments.

Even though I think that the manuscript is almost ready for publication, I would only like that the authors clarify the balanced vs. imbalanced dataset analyses and conclusions.

Training with an imbalanced dataset can lead to low accuracy for under-represented classes because of the lack of examples to learn from. This is well illustrated in the leave-one-order-out analyses in which 2 out of 23 orders were under-performed when not present in the training dataset (table 4S). Even though the authors seem to depreciate this finding (“only 2 out of 23 orders were significantly worse when not having images from these orders in the training set.” in their reply; and it is not well explain the practical implications of this in the discussion). This finding shows exactly the limitation of not having a good representation of all orders in the dataset: that the average (across all orders in an imbalanced test dataset) performance of the model might not necessarily be extrapolated to all types of data that are not present (or are under-represented) in the training and test dataset. In lines 690-697 the authors address this result, however, they only present potential solutions (e.g. data augmentation), but ultimately they should also point out that it is not a good idea to blindly predict points for a specific order that was under-represented (or absent) in the training and/or test dataset.

We apologise that in our last response, we didn’t emphasize our findings on the leave-one-order-our analysis. We rewrote this part of the discussion (Line 689 – 711 for the highlighted version) about (1) the impact of missing orders, (2) the possible causes for the remaining 21 orders that are not having worse results and (3) how to deal with absent or underrepresented groups. In particular we draw attention to the issue of poor predictions for underrepresented groups, even when the average prediction accuracy is high and provide a note of caution for researchers (Line 703 – 711 for the highlighted version).

Another important point that is not clear regarding imbalanced datasets is the misinterpretation of the evaluation of the model’s performance if the test dataset is imbalanced. For example, in the results it is stated that “the pixel distance between expert and predicted points averaged 47.3 (1.4% of the image height – 3280 pixels) across all 15 points (10 body regions and 5 reflectance standards)” (line 456-457). However, if the test dataset is not balanced and instead it is representative of the imbalanced full dataset this value of 1.4% would be mostly driven by the performance of the model in predicting points for Passeriformes (as this represents 60% of the data). This means that one cannot use your trained model to predict points for, for example, Leptosomiformes and expect an error of 1.4%. This has to be clearly explained in the discussion.

We discuss how our model would perform on new bird photos. We think that it would perform well on well-represented groups, and also address that the performance would be limited for underrepresented groups. We also add the discussion of the generalisation of our model on other datasets. (Line 712 – 722 for the highlighted version).

Finally, while I think that the analyses comparing a balanced and imbalanced training datasets and evaluate the performance of the resulting models on a balanced test dataset is a very useful illustration of the problem of imbalanced datasets, I do not agree that showing the inverse (i.e. evaluate on an imbalanced dataset) is useful at all and can actually mislead the readers. The ultimate goal of training a model to perform a task is to perform this task well across all the possible types of data and not to obtain a “good value” of a given evaluation metric (that might result from an imbalanced test dataset and be manly driven by a handful of data types, in this case orders). Having a model that performs well across all types of data is what can avoid a systematic bias in our predicted data and make your model generalisable (e.g. imagine that someone wants to use your model just for Leptosomiformes, the performance of the model for Passeriformes will be irrelevant). Evaluating a model trained on an imbalanced dataset and tested on an imbalanced dataset does not show that is better to use this strategy, it only shows that the model will perform better with more training data (e.g. if Passeriformes are 60% of the data in both training and test datasets, the model has many data to learn from for this order and the evaluation metric will be manly driven by this order alone, and obviously result in a “good model”). While creating a balanced training dataset might in some cases be difficult without excluding some data for the most common orders (which is not the ideal thing to do and unlikely to be a good advice for most situations) and therefore be better to train with an imbalanced training dataset, it is hard to justify testing the model on an imbalanced dataset if the final aim is to generalize the model across all orders. I would therefore remove any analyses that use the imbalanced dataset for evaluation of the models’ performances as this can be misleading to the readers.

We appreciate the reviewer’s comments on the imbalanced dataset. However, the main reason for us to include the imbalanced dataset is to address whether, for a full dataset that is imbalanced (e.g. different numbers of specimens per order), a balanced (equal numbers of specimens per group) training set performs differently from an imbalanced training set (where the imbalance in the training set is representative of the overall imbalance in the data)?

We think this is relevant because there will be use cases, similar to our own, where a full dataset is imbalanced and researchers want to use DL to predict all images. So we want to see with the same training set size if an imbalanced training set would perform as accurately as a balanced training set. If an imbalanced training set performs well (and specifically improves predictions for the poorly representative groups), it has an advantage as it can have a larger training set size. Take our dataset as an example, it only has 54 images from the Eurypygiformes order, which it’s impossible to make a training set with enough images from Eurypygiformes. And making a training set that has enough images is important, as the number of training images would affect the model’s performance.

Using an imbalanced and representative training set can do a good job based on our results. Although predictions should be more accurate on majority orders and less accurate on underrepresented orders, we can say the predictions are accurate based on the evaluation metric (the overall pixel distance). In addition, we mentioned in the discussion that researchers should pay more attention to underrepresented groups. If a dataset has images from underrepresented groups, and researchers are concerned about the performance of those groups (e.g. low accuracy in the validation images), they can exclude those groups from the full dataset and treat them separately (e.g. manually label or train a model separately).

Taken together, we suggest using a train set that represents well to the full dataset as an initial and global way to create training sets, which can prevent the training set is too small and maintain accuracy. While we also pointed out that it’s important to keep an eye on underrepresented groups.

Other minor comments:

Line 107 I think that “automated point annotation” sound better as annotation often refers to annotating the data to create a dataset to train a deep learning model.

We agreed with the reviewer’s comment and update the phrase to “automated point annotation”.

Line 186 each specimen was only photographed once right? You shouldn’t have the same specimen in the training and test dataset as this will contribute to a higher overall accuracy, but not necessarily represent the generalization capability of the model to predict points in unseen specimens (i.e. it is easier to predict points on a new image from a specimen present in the training dataset than on a image from a completely new specimen). If you used the same specimens on the training and test dataset you should clearly state that and explain why you did not keep them separate.

We clarify that specimens were only either in the training or test set. (line 258 – 264 for the highlighted version)

Shouldn’t all tables that show results per order also include a column in which the pixel distances were corrected for the size of the pictures (e.g. % of the image height) so that we could also compare the error between the different orders?

We have updated the S3 Table and S4 Table by adding the pixel distances and the equivalent proportion of image heights.
